# Association between preoperative interruption of antiplatelet therapy and postoperative thrombotic risk after minimally invasive surgery for abdominopelvic cancer in patients treated with P2Y12 inhibitors

**DOI:** 10.1186/s12916-026-04634-0

**Published:** 2026-01-16

**Authors:** Masashi Kubota, Satomi Yoshida, Takayuki Goto, Toshiki Fukasawa, Takayuki Anno, Gaku Fujiwara, Satoshi Toshiyama, Yoshihide Inayama, Takanori Yanai, Takayuki Sumiyoshi, Ryoichi Saito, Takashi Kobayashi, Koji Kawakami

**Affiliations:** 1 Department of Pharmacoepidemiology, Graduate School of Medicine and Public Health, Kyoto University, Yoshidakonoecho, Sakyo-ku, Kyoto, 606-8501 Japan; 2 Department of Urology, Graduate School of Medicine, Kyoto University, Kyoto, Japan; 3 Department of Clinical Medicine, Institute of Medicine, University of Tsukuba, Tsukuba, Japan; 4 Department of Gynecology and Obstetrics, Graduate School of Medicine, Kyoto University, Kyoto, Japan

**Keywords:** P2Y12 inhibitor, Non-cardiac, Clopidogrel, Robot, Laparoscopic, Cancer

## Abstract

**Background:**

The safety of preoperative discontinuation of antiplatelet therapy for arterial thrombotic complications in non-cardiac, high-bleeding-risk surgery among patients receiving P2Y12 inhibitors remains poorly understood. This study compares the effect of preoperative discontinuation of antiplatelet therapy versus maintenance on thrombotic complications in patients receiving P2Y12 inhibitors who undergo minimally invasive surgery (MIS) for abdominal or pelvic cancer.

**Methods:**

In this cohort study, we identified patients receiving P2Y12 inhibitors who underwent planned MIS for abdominopelvic cancer from two hospital-based databases. They were divided into an interruption (exposure) group that discontinued antiplatelet therapy 5 days before surgery and a maintenance (control) group that continued therapy based on confirmed prescriptions less than 5 days before surgery. After adjusting for confounders, we evaluated the weighted risk ratios (RRs) for postoperative interventions over 90 days.

**Results:**

A total of 1365 eligible patients were divided into an interruption group (*n* = 1157) and a maintenance group (*n* = 208). The interruption group had increased risk of thrombotic complications (RR, 3.29; 95% confidence interval (CI), 1.15 to 9.41), particularly in postoperative coronary artery disease (RR, 5.30; 95% CI, 1.27 to 22.12). This elevated risk was notable among patients receiving dual antiplatelet therapy preoperatively. The interruption group did not show increased risk of postoperative ischemic stroke or peripheral arterial disease, nor did it substantially differ in hemostasis procedures, blood transfusions, or mortality. Notably, patients who discontinued antiplatelet therapy exhibited a higher overall risk of vascular events (RR, 2.54; 95% CI, 1.16 to 5.56).

**Conclusions:**

Discontinuing antiplatelet therapy in patients receiving P2Y12 inhibitors more than 5 days before MIS was associated with an increased risk of postoperative coronary artery disease, without providing any benefit in terms of bleeding control or mortality.

**Supplementary Information:**

The online version contains supplementary material available at 10.1186/s12916-026-04634-0.

## Background

Minimally invasive surgery (MIS), including pure laparoscopic and robot-assisted approaches, has recently become the standard for the radical treatment of abdominopelvic solid tumors, such as urologic, gastrointestinal, and gynecological cancers [1–7]. An increasing number of patients undergoing radical surgery to control cancer progression have a history of arterial thrombotic disease (e.g., coronary artery disease (CAD), ischemic stroke, and peripheral arterial disease (PAD)) [8–10]. Because most of these patients require antiplatelet therapy, MIS has gained popularity for its superior perioperative bleeding control [11–13].

P2Y12 inhibitors, a relatively new class of antiplatelet agents, have proven effective for the secondary prevention of arterial thrombotic disease [14–17]. However, their use also increases the risk of hemorrhagic complications [18–20]. Additionally, surgeries for abdominopelvic solid tumors are often classified as high-bleeding-risk procedures [21, 22]. After interruption of aspirin (acetylsalicylic acid; ASA) or the P2Y12 inhibitors clopidogrel and prasugrel to reduce the risk of hemorrhagic complications, platelet function generally takes 7–10 days to recover, because these drugs irreversibly inhibit platelet activity and recovery depends on the replacement of affected platelets over their lifespan [23]. However, previous observational data indicate that interruption for more than 5 days is associated with a higher incidence of major adverse cardiovascular events [24, 25]. Reflecting this balance between bleeding and thrombotic risks, current guidelines recommend a preoperative discontinuation period of 5 days for clopidogrel and 7 days for prasugrel before non-cardiac, high-bleeding-risk surgeries [21]. For patients at high thrombotic risk, bridging with ASA during surgery has also been suggested [21, 22, 26, 27]. Nonetheless, no prospective studies have been conducted, meaning definitive guidance is lacking on the management of patients receiving P2Y12 inhibitors who undergo non-cardiac surgeries [28]. Previous studies have primarily focused on bleeding complications associated with continuing P2Y12 inhibitors during non-cardiac cancer surgeries [29, 30]. In contrast, data remain limited on the safety of preoperative discontinuation of P2Y12 inhibitors; this limitation is partly due to small sample sizes and the low incidence of severe complications, and particularly impacts arterial thrombotic events in specific non-cardiac surgeries.

We hypothesized that focusing on MIS—known for its superior bleeding control and potential to permit the continuation of antiplatelet therapy—would clarify the thrombotic risks associated with P2Y12 inhibitor discontinuation. Here, we used large-scale observational data to compare the effects of preoperative discontinuation versus maintenance of antiplatelet therapy on postoperative thrombotic complications during minimally invasive radical surgery for abdominal or pelvic cancers in patients receiving P2Y12 inhibitors.

## Methods

### Data sources

We used two Japanese hospital-based databases, the JMDC hospital database, maintained by JMDC Inc., and the RWD database, maintained by the Health, Clinic, and Education Information Evaluation Institute (HCEI) under the auspices of JMDC Inc. [31–33] As of 2024, the JMDC hospital database included data on over 38.0 million in- and outpatients at 1001 acute and sub-acute care hospitals, while the RWD database contained data on 14.3 million inpatients and outpatients at 220 clinics and hospitals. These databases collect information from electronic medical records (EMRs), claims, and the Diagnosis Procedure Combination (DPC) system, [34] including demographics, diagnoses, procedures, and medications. Because DPC data contain specific information on surgery type and oncological background, we restricted data collection to DPC hospitals that could provide these details. Data for this study were extracted from the JMDC hospital database from April 2014 to January 2024 and from the RWD database from April 2006 to May 2024. The extracted data were merged into a single dataset for analysis according to the previous study [35], as both provided compatible information on the variables necessary for this study. To prevent biased estimates resulting from overlap of medical institutions, data from the JMDC hospital database were used for those institutions exclusively.

### Study population

We identified patients who underwent laparoscopic or robot-assisted radical resection of a single organ for cancer after October 2016, the date marking the nationwide standardization of transcribing patient-brought medications into hospital orders. Eligible cancers included prostate, kidney, bladder, upper urinary tract urothelial, rectal, stomach, colon, liver, pancreatic, bile duct, endometrial, and cervical cancers. We also required that patients had been prescribed antiplatelet agents, including at least one P2Y12 inhibitor (clopidogrel or prasugrel), at the surgical institution for at least 28 days before surgery. Ticlopidine and ticagrelor were excluded because their use is limited in Japan and their recommended preoperative discontinuation periods differ. [36–39].

We excluded patients who (i) received anticoagulant therapy (e.g., direct oral anticoagulants or warfarin) from 28 days before surgery to the date of surgery, (ii) underwent surgery for recurrent or metastatic cancer, (iii) were primarily admitted for a non-cancer-related condition (e.g., infection or anemia), (iv) had comorbid thrombocytopenia or liver cirrhosis on admission, or (v) were treated at hospitals that ceased providing data to the databases during the follow-up period. The coding definitions for these inclusion and exclusion criteria are presented in Additional file 1: Table S1.

### Exposure

Study exposure was defined as the discontinuation of antiplatelet therapy at least 5 days before surgery. This discontinuation, recommended for clopidogrel, also encompasses the recommended 7 days for prasugrel and was applied uniformly to all P2Y12 inhibitors to examine perioperative management strategies in this broader patient population. The interruption group (exposure) was identified by confirming the absence of antiplatelet therapy in medication records during that period. Conversely, the maintenance group (unexposed reference) was defined as having at least one in-hospital prescription for antiplatelet agents from 4 days before surgery until the day of surgery, and presumably retained antiplatelet effects throughout the operation. Patients who had been receiving P2Y12 inhibitors before admission but were switched to ASA or cilostazol alone, or for whom the P2Y12 component was withheld from dual antiplatelet therapy (DAPT), were also classified as maintenance. Maintenance or discontinuation of P2Y12 inhibitor therapy were determined at the discretion of the treating physicians, based on each patient’s thrombotic and bleeding risk profiles and institutional policies regarding perioperative antiplatelet management.

### Outcomes

The primary outcome was the incidence of therapeutic interventions for thrombotic complications—CAD, ischemic stroke, and PAD—within the first 90 days postoperatively. CAD was defined by a combination of diagnostic codes and procedural interventions, including percutaneous coronary intervention (PCI) or coronary artery bypass grafting (CABG). Ischemic stroke was defined by a combination of diagnostic codes and procedural interventions, including endovascular thrombectomy, intra-arterial thrombolysis, intracranial percutaneous transluminal angioplasty or stenting, and pharmacological interventions involving the intravenous administration of agents, such as edaravone, argatroban, ozagrel (a selective thromboxane A2-synthetase inhibitor), urokinase, or alteplase. PAD was defined by a combination of diagnostic codes and procedural interventions, including vascular dilatation and thrombectomy of the affected limb arteries.

Secondary outcomes included (i) overall vascular events (composite of thrombotic and bleeding events requiring intervention), (ii) bleeding, and (iii) overall mortality within 90 days postoperatively. Bleeding was evaluated based on the incidence of therapeutic interventions for hemorrhagic complications, defined as the need for hemostatic procedures or allogeneic blood transfusions. Hemostatic procedures included angiographic embolization, open or laparoscopic surgery for hemorrhage control, endoscopic gastrointestinal hemostasis, endoscopic hemostasis for the small and large intestines, transurethral electrocoagulation, and transurethral renal pelvic or ureteral coagulation. The coding definitions for these outcomes are presented in Additional file 1: Table S2.

### Covariates

Patient characteristics were assessed during the baseline period before surgery. Based on previous studies [19, 30, 35, 40], we selected the following covariates: demographics (age, sex); BMI (category); smoking history (defined as > 20 pack-years); hospital size (< 500, ≥ 500 beds); surgical type and approach (pure laparoscopy or robot-assisted); medication-related covariates (the type and dosage of P2Y12 receptor inhibitors, and the number [DAPT or triple antiplatelet therapy] and the type [ASA and cilostazol] of combined antiplatelet therapy); and clinical factors, including locally advanced cancer (cT3, cN1, or higher), CAD status (categorized as post-PCI, post-CABG, medication-only, or no history), history of ischemic stroke, history of arteriosclerosis obliterans or extracranial arterial stenosis, and the presence of diabetic complications (retinopathy, nephropathy, or neuropathy). The coding definitions for these covariates are presented in Additional file 1: Table S3.

### Statistical analysis

Missing data were addressed using multiple imputation with 100 datasets under the assumption of data missing at random. We adjusted for confounders using inverse probability treatment weighting (IPTW), a propensity score method [41]. Propensity scores for the interruption of P2Y12 inhibitor therapy were calculated using a multivariable logistic regression model which included the covariates listed above. These scores were then used to derive stabilized weights, which were trimmed at the 99th percentile to mitigate the influence of extreme weights. Covariate balance before and after IPTW was evaluated using standardized mean differences (SMDs), with values < 10% considered well balanced. We used weighted modified Poisson regression and weighted linear binomial regression models, both with robust variance estimators, to estimate risk ratios (RRs) and risk differences (RDs), along with their 95% confidence intervals (CIs).

We conducted two sensitivity analyses to confirm the robustness of our findings. First, we redefined the maintenance group (unexposed reference) more strictly, requiring antiplatelet medication prescriptions up to 1 day before or on the day of surgery. Second, we conducted a complete-case analysis by excluding any cases with missing data.

We also performed seven subgroup analyses to assess effect modification by the following factors: (i) CAD history; (ii) ischemic stroke history; (iii) PAD history; (iv) administration of DAPT with a P2Y12 inhibitor 28 days before surgery; (v) administration of SAPT with a P2Y12 inhibitor 28 days before surgery; (vi) pelvic surgery; and (vii) abdominal surgery. For each subgroup analysis, we re-estimated propensity scores and re‐performed IPTW.

We additionally computed E-values to assess the robustness of our findings to unmeasured confounding [42, 43]. E-values were calculated for point estimates and the lower bounds of the 95% confidence intervals.

In addition to the predefined analyses, we conducted an exploratory analysis to evaluate the temporal pattern of thrombotic complications. We estimated IPTW-weighted cumulative incidence functions for thrombotic complications, treating death as a competing risk.

All statistical analyses were performed using R version 4.3.2 (R Foundation for Statistical Computing, Vienna, Austria). The R codes for the primary analysis are available in Additional file 1: Appendix 1.

## Results

### Description of the study population

**Fig. 1:**
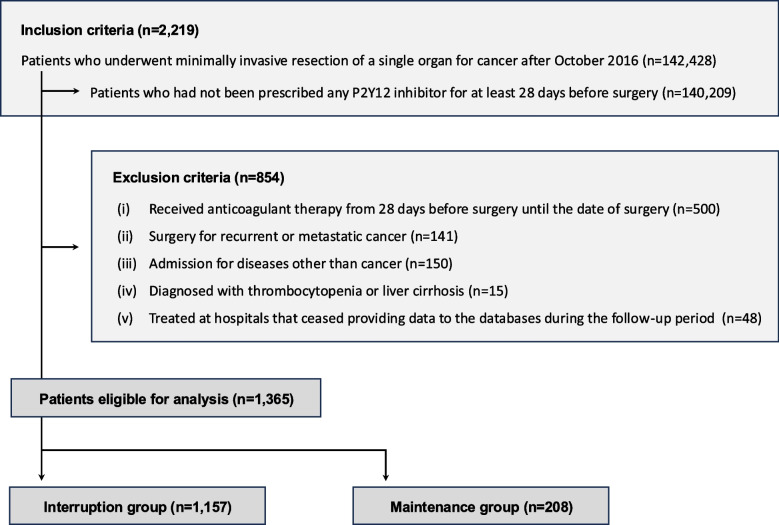
Flow diagram of the study

A total of 1365 patients were eligible for this study, consisting of 1157 in the interruption group and 208 in the maintenance group (Fig. 1). Details of missing data among these patients are provided in Additional file 1: Table S4.

Baseline characteristics are shown in Table 1. In the maintenance group, 54.8% of patients received a P2Y12 inhibitor (38.5% received P2Y12 inhibitor monotherapy and 15.9% received DAPT), and 45.2% received ASA or cilostazol monotherapy during the 4 days preceding surgery. No patients who underwent pancreaticoduodenectomy were included in either group. Before IPTW, the maintenance group had a higher proportion of patients who underwent surgery at high-volume centers (≥ 500 beds), who opted for robot-assisted surgery, and who were undergoing DAPT with ASA for their underlying diseases. In contrast, the interruption group had a higher prevalence of patients with a history of ischemic stroke and an older median age. After IPTW, covariates were well balanced, with SMDs below 10% (Additional file 1: Fig. S1 and S2).

**Table 1:** Baseline characteristics of the interruption and maintenance groups before and after IPTW

Characteristic	Overall (*n* = 1365)	Before IPTW			After IPTW^a^		
		**Interruption group** **(*n* = 1157)**	**Maintenance group** **(*n* = 208)**	**SMD**	**Interruption group** **(*n* = 1364)**	**Maintenance group** **(*n* = 1197)**	**SMD**
Age, median (IQR)	75.0 (70, 81)	75.0 (70, 80)	75.0 (69, 82)	0.103	75 (70, 80)	76 (70, 82)	−0.021
Men, *n* (%)	1,092 (80.0)	927 (80.1)	165 (79.3)	0.008	1,093 (80.1)	953 (79.6)	0.006
BMI, median (IQR)	23.2 (21.1, 25.3)	23.2 (21.1, 25.2)	22.9 (21.1, 25.8)	− 0.041	23.2 (21.0, 25.2)	22.5 (20.3, 25.6)	0.057
Smoking, pack-years ≥ 20, *n* (%)	670 (49.1)	561 (48.5)	109 (52.4)	− 0.039	669 (49)	572 (47.8)	0.013
Hospital size, beds ≥ 500, *n* (%)	610 (44.7)	448 (38.7)	162 (77.9)	− 0.392	608 (44.6)	577 (48.2)	− 0.036
Clinical T ≥ 3, or N ≥ 1 cancer, *n* (%)	633 (46.4)	552 (47.7)	81 (38.9)	0.088	635 (46.6)	547 (45.7)	0.008
History of arterial vascular disease, *n* (%)							
CAD without PCI, or CABG	653 (47.8)	559 (48.3)	94 (45.2)	0.031	659 (48.3)	674 (56.3)	− 0.081
Post-PCI	313 (22.9)	251 (21.7)	62 (29.8)	− 0.081	310 (22.7)	254 (21.2)	0.015
Post-CABG	23 (1.7)	20 (1.7)	3 (1.4)	0.003	23 (1.7)	24 (2.0)	− 0.003
Ischemic stroke	681 (49.9)	611 (52.8)	70 (33.7)	0.192	681 (49.9)	546 (45.6)	0.043
PAD	583 (42.7)	500 (43.2)	83 (39.9)	0.033	586 (43.0)	556 (46.4)	− 0.035
History of diabetic complications, *n* (%)	267 (19.6)	237 (20.5)	30 (14.4)	0.061	267 (19.6)	207 (17.3)	0.022
Daily P2Y12 inhibitor type and dose, *n* (%)							
Clopidogrel 25 mg	25 (1.8)	25 (2.2)	0 (0)	0.022	25 (1.8)	0 (0)	0.018
Clopidogrel 50 mg	17 (1.2)	15 (1.3)	2 (1.0)	0.003	17 (1.2)	16 (1.3)	− 0.001
Clopidogrel 75 mg	1,115 (81.7)	952 (82.3)	163 (78.4)	0.039	1,114 (81.7)	973 (81.3)	0.005
Prasugrel 3.75 mg	208 (15.2)	165 (14.3)	43 (20.7)	− 0.064	208 (15.2)	208 (17.4)	− 0.022
Number of daily combined antiplatelets, *n* (%)							
DAPT	655 (48)	523 (45.2)	132 (63.5)	− 0.183	656 (48.1)	686 (57.3)	− 0.092
TAPT	62 (4.5)	46 (4.0)	16 (7.7)	− 0.037	64 (4.7)	73 (6.1)	− 0.014
Type of daily combined antiplatelets, *n* (%)							
ASA	628 (46.0)	497 (43.0)	131 (63.0)	− 0.200	630 (46.2)	653 (54.6)	− 0.083
Cilostazol	150 (11.0)	118 (10.2)	32 (15.4)	− 0.052	153 (11.2)	175 (14.6)	− 0.034
Robot-assisted surgery, *n* (%)	155 (11.4)	108 (9.3)	47 (22.6)	− 0.133	149 (10.9)	112 (9.4)	0.016
Surgery type, *n* (%)							
Colectomy, sigmoidectomy	523 (38.3)	450 (38.9)	73 (35.1)	0.038	523 (38.3)	462 (38.6)	− 0.003
Distal gastrectomy	128 (9.4)	102 (8.8)	26 (12.5)	− 0.037	132 (9.7)	164 (13.7)	− 0.040
Distal pancreatectomy	6 (0.4)	4 (0.3)	2 (1.0)	− 0.006	6 (0.4)	5 (0.4)	0.001
Hepatic resection	21 (1.5)	14 (1.2)	7 (3.4)	− 0.022	21 (1.5)	18 (1.5)	0.000
Hepatic segmentectomy	9 (0.7)	7 (0.6)	2 (1.0)	− 0.004	9 (0.7)	6 (0.5)	0.001
Partial nephrectomy	6 (0.4)	6 (0.5)	0 (0)	0.005	6 (0.4)	0 (0)	0.004
Proximal gastrectomy	80 (5.9)	78 (6.7)	2 (1.0)	0.058	80 (5.9)	27 (2.3)	0.036
Radical prostatectomy	93 (6.8)	69 (6.0)	24 (11.5)	− 0.056	91 (6.7)	65 (5.4)	0.012
Rectal amputation	39 (2.9)	34 (2.9)	5 (2.4)	0.005	38 (2.8)	30 (2.5)	0.003
Rectal resection	226 (16.6)	198 (17.1)	28 (13.5)	0.037	224 (16.4)	171 (14.3)	0.021
Total cystectomy	11 (0.8)	8 (0.7)	3 (1.4)	− 0.008	11 (0.8)	7 (0.6)	0.002
Total gastrectomy	36 (2.6)	29 (2.5)	7 (3.4)	− 0.009	38 (2.8)	70 (5.8)	− 0.031
Total hysterectomy	4 (0.3)	1 (0.1)	3 (1.4)	− 0.014	3 (0.2)	4 (0.3)	− 0.001
Total nephrectomy	21 (1.5)	14 (1.2)	7 (3.4)	− 0.022	19 (1.4)	13 (1.1)	0.003
Total nephroureterectomy	162 (11.9)	143 (12.4)	19 (9.1)	0.032	162 (11.9)	153 (12.8)	− 0.008
Maintained antiplatelets during surgery, *n* (%)							
P2Y12 inhibitor alone	80 (5.9)	0 (0)	80 (38.5)	NA	0 (0)	496 (41.4)	NA
DAPT	33 (2.4)	0 (0)	33 (15.9)		0 (0)	153 (12.8)	
TAPT	1 (0.1)	0 (0)	1 (0.5)		0 (0)	4 (0.3)	
ASA alone	82 (6.0)	0 (0)	82 (39.4)		0 (0)	443 (37.0)	
Cilostazol alone	12 (0.9)	0 (0)	12 (5.8)		0 (0)	102 (8.5)	

*ASA *acetylsalicylic acid, *BMI *body mass index, *CABG *coronary artery bypass graft, *CAD *coronary artery disease, *DAPT *dual antiplatelet therapy, *IQR *interquartile range, *PAD *peripheral arterial disease, *PCI *percutaneous coronary intervention, *SMD *standardized mean difference, *TAPT *triple antiplatelet therapy

^a^Frequency numbers were rounded to integers based on weight. Adjusted for age, sex, BMI category, smoking status, hospital size, invasive cancer, history of CAD (medication only), post-PCI, post-CABG, ischemic stroke, PAD, diabetic complication, type and dosage of P2Y12 receptor inhibitors, number (DAPT or TAPT), and type (ASA and cilostazol) of combined antiplatelet therapy, robot-assisted surgery, and surgery type

### Risk of thrombotic events

**Fig. 2:**
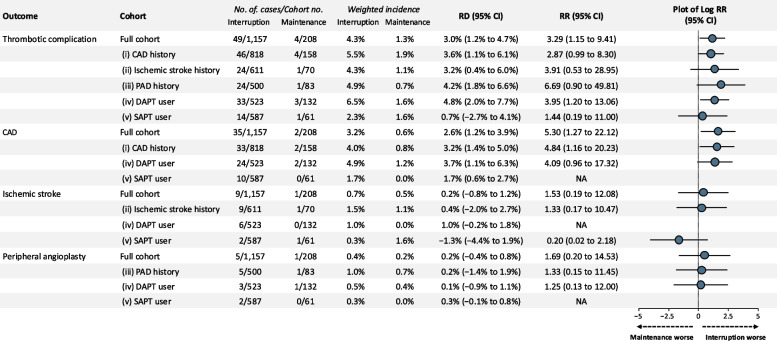
Risk of thrombotic events. This figure presents the case number of thrombotic events, weighted incidence, risk ratios, risk differences, and forest plots of the log risk ratios for the primary outcome. For each outcome, a row showing results for the full cohort is displayed, followed by rows for various subgroup analysis results. Abbreviations: CAD, coronary artery disease; CI, confidence interval; DAPT, dual antiplatelet therapy; NA, not available; SAPT, single antiplatelet therapy; PAD, peripheral arterial disease; RD, risk difference; RR, risk ratio

Figure 2 shows the weighted incidence, RRs, and RDs for thrombotic outcomes. Overall, this study showed an increased risk of secondary interventions for thrombotic complications and CAD. The weighted incidences of overall thrombotic complications were 4.3% in the interruption group and 1.3% in the maintenance group. Comparing the interruption group with the maintenance group, the weighted RRs and RDs for the full cohort were 3.29 (95% CI, 1.15 to 9.41) and 3.0% (95% CI, 1.2% to 4.7%) for overall thrombotic complications; and 5.30 (95% CI, 1.27 to 22.12) and 2.6% (95% CI, 1.2% to 3.9%) for interventions for CAD by PCI or CABG. However, interruption did not increase the risk of ischemic stroke or PAD. Furthermore, none of the sensitivity analyses yielded substantial changes in the effect estimates (Additional file 1: Fig. S3). Among thrombotic outcomes, overall thrombotic complications and interventions for CAD were associated with increased risk in the interruption group compared with that in the maintenance group.

In the subgroup analyses, a history of CAD was associated with increased risk of exacerbation of underlying disease in the interruption group. The weighted RRs and RDs were 4.84 (95% CI, 1.16 to 20.23) and 3.2% (95% CI, 1.4% to 5.0%) for interventions for CAD by PCI or CABG. The administration of DAPT was also associated with increased risk of secondary interventions for thrombotic complications. The weighted RRs and RDs for these were 3.95 (95% CI, 1.20 to 13.06) and 4.8% (95% CI, 2.0% to 7.7%) (Fig. 2 and Additional file 1: Fig. S4). Subgroup analyses by surgical site, specifically pelvic surgery and abdominal surgery, did not show any notable differences in RRs or RDs for the outcomes (Additional file 1: Fig. S5).

For overall thrombotic complications (RR 3.29, 95% CI, 1.15 to 9.41), the *E*-value was 6.03 for the point estimate and 1.57 for the lower CI bound. For CAD interventions (RR 5.30, 95% CI, 1.27 to 22.12), the corresponding *E*-values were 10.07 and 1.86, respectively (Fig. 2; Additional file 1: Fig. S6).

**Fig. 3:**
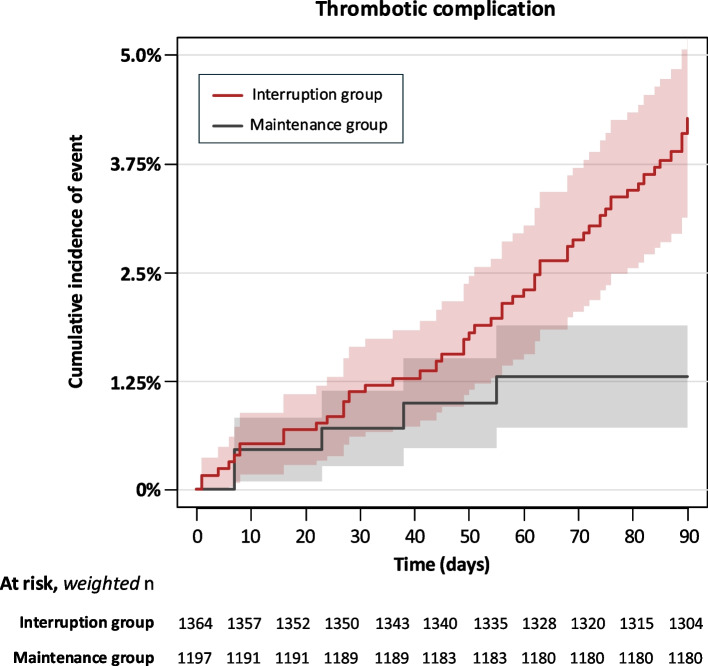
IPTW-weighted cumulative incidence function for thrombotic complications, with death as the competing event

In an exploratory analysis, IPTW-weighted cumulative incidence curves for thrombotic complications were plotted (Fig. 3). The curves revealed that the interruption group consistently exhibited a higher risk of thrombotic complications throughout the 90-day postoperative period.

**Fig. 4:**
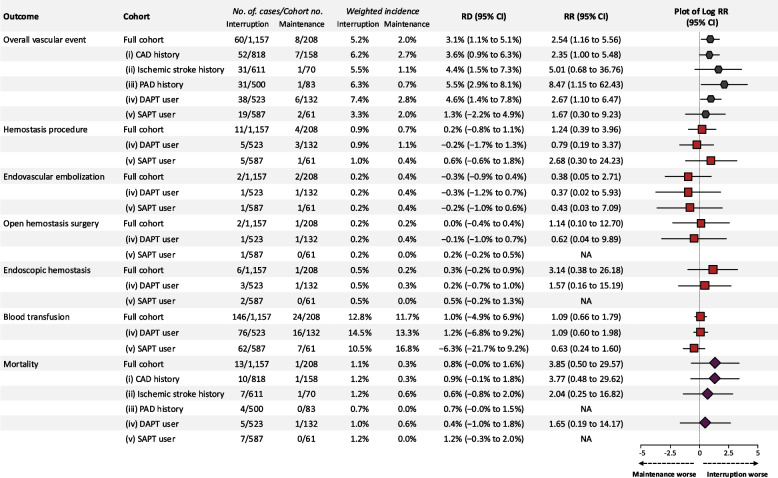
Risk of overall vascular events, bleeding events and mortality. This figure presents the numbers of overall vascular events (a composite of thrombotic and bleeding events requiring intervention), bleeding events, and mortality, along with the weighted incidence, risk ratios, risk differences, and forest plots of the log risk ratios for secondary outcomes. Abbreviations: CI, confidence interval; DAPT, dual antiplatelet therapy; NA, not available; SAPT, single antiplatelet therapy; RD, risk difference; RR, risk ratio

### Risk of overall vascular events, bleeding, and mortality

Figure 4 illustrates the outcomes for overall vascular events (a composite of thrombotic and bleeding events requiring intervention), bleeding, and overall mortality. The risk of overall vascular events was elevated not only in the main analysis (RR, 2.54; 95% CI, 1.16 to 5.56), for which the corresponding *E*-value was 4.52 for the point estimate and 1.59 for the lower CI bound (Additional file 1: Fig. S6), but was also observed in subgroup analyses, including patients with a history of CAD (RR, 2.35; 95% CI, 1.00 to 5.48), PAD (RR, 8.47; 95% CI, 1.15 to 62.43), and those receiving DAPT (RR, 2.67; 95% CI, 1.10 to 6.47). Interruption of P2Y12 inhibitor therapy did not reduce the risk of bleeding events or mortality. The respective weighted incidences of overall hemostasis procedures, blood transfusion, and overall mortality were 0.9%, 12.8%, and 1.1% in the interruption group versus 0.7%, 11.7%, and 0.3% in the maintenance group. Comparing the two groups, the weighted RRs and RDs for the entire cohort were 1.24 (95% CI, 0.39 to 3.96) and 0.2% (95% CI, − 0.8% to 1.1%) for overall hemostasis procedures; 1.09 (95% CI, 0.66 to 1.79) and 1.0% (95% CI, − 4.9% to 6.9%) for blood transfusions; and 3.85 (95% CI, 0.50 to 29.57) and 0.8% (95% CI, 0% to 1.6%) for mortality, respectively. Exposure to interruption did not increase the risk of bleeding or other outcomes in any of the sensitivity analyses (Additional file 1: Fig. S3). When the maintenance group was redefined more strictly as having antiplatelet prescriptions up to 1 day before or on the day of surgery, the risks of overall hemostasis procedures and blood transfusion remained unchanged (1.54 [95% CI, 0.42 to 5.63] and 1.37 [95% CI, 0.84 to 2.25], respectively), consistent with the main analysis. Furthermore, no subgroup exhibited a lower risk of bleeding or mortality in the interruption group compared with the maintenance group (Fig. 4 and Additional file 1: Fig. S4).

## Discussion

Our findings indicated that discontinuing antiplatelet therapy for more than 5 days before planned MIS for abdominopelvic cancer in patients receiving P2Y12 inhibitors increased the risk of postoperative thrombotic complications, particularly CAD. Moreover, maintaining therapy did not increase the risk of postoperative bleeding events or mortality. Notably, discontinuation was associated with a higher overall risk of vascular events.

To our knowledge, this is the first study to investigate the effect of preoperative discontinuation of antiplatelet therapy in P2Y12 inhibitor users on postoperative thrombotic events following MIS for abdominopelvic cancer. A previous Cochrane review reported that continuing or discontinuing antiplatelet therapy (including ASA and P2Y12 inhibitors) at least 5 days before non-cardiac surgery appeared to have little or no impact on ischemic events, bleeding requiring surgical intervention, or mortality [44]. However, in patients treated with clopidogrel for CAD, the incidence of cardiovascular adverse events rises substantially within 90 days after clopidogrel discontinuation [45, 46]. A recent prospective observational study focusing on gastroenterological surgeries also indicated that preoperative aspirin discontinuation substantially increased the incidence of postoperative thrombotic events [45]. Consistent with these findings, our results suggest that discontinuing antiplatelet therapy before abdominopelvic MIS in patients receiving P2Y12 inhibitors may elevate thrombotic risk and, in turn, may emphasize the need for caution in managing these patients. Notably, CAD had the highest incidence among postoperative thrombotic events, strongly influencing the overall thrombotic complications. This finding warrants careful interpretation of our results. Our cumulative incidence curves indicated that patients in the interruption group consistently exhibited a higher risk of thrombotic complications throughout the 90-day postoperative period, highlighting the need for vigilance beyond the acute perioperative phase. However, further studies incorporating information on the timing of postoperative resumption of P2Y12 inhibitors are warranted to better clarify the clinical implications.

Our analyses revealed that the maintenance of antiplatelet therapy during MIS did not increase the risk of severe hemorrhagic complications requiring secondary anesthetic procedures or of mortality. This finding is consistent with previous studies [44–46]. Generally, cancer surgeries for solid tumors have a 30-day risk of major bleeding exceeding 2%, classifying them as high-bleeding-risk procedures [21]. However, MIS for abdominopelvic malignancies has been associated with lower transfusion and complication rates than with open surgery [1, 2, 4–6, 47]. The incidence of major bleeding complications is estimated at 0.3–1.0% among patients who continued ASA therapy through surgery [30, 45, 46]. In our analysis, the weighted incidence of major bleeding requiring hemostatic procedures was only 0.8% in the cohort that maintained antiplatelet therapy through surgery, including the 54.8% who continued P2Y12 inhibitors. In various non-cardiac surgeries, recent surgical advancements have decreased bleeding risk to a level below thrombotic risk, even for procedures typically classified as high-bleeding-risk. This finding emphasizes the need to refine perioperative management guidelines for antiplatelet therapy, tailored to specific surgical interventions.

A key strength of our study is its use of large-scale data to assess outcomes related to rare yet severe postoperative thrombotic events in P2Y12 inhibitor users. Because the exposure definition was based on prescription records within claims rather than on free-text electronic health records, potential reporting errors were unlikely to affect the classification of exposure status. These results serve to caution clinicians against automatic adherence to uniform recommendations for the preoperative discontinuation of antiplatelet therapy. In real-world settings, patients often consult specialists regarding thrombosis and P2Y12 inhibitor therapy before surgery; accordingly, the maintenance group likely included patients at high thrombotic risk for whom discontinuation was deemed impractical, whereas the interruption group likely comprised those deemed clinically safe to discontinue therapy. This indicates that the observed interruption effect on thrombosis may have been conservative, even after IPTW using many covariates. Alternatively, a substantial proportion of truly high-thrombotic-risk patients may currently be erroneously considered suitable for preoperative antiplatelet discontinuation, which could result in preventable postoperative thrombotic events. Our findings may help guide clinical decision-making on P2Y12 inhibitor management in similar clinical contexts, particularly for planned radical surgeries for non-metastatic primary cancers.

This study has several limitations. First, unmeasured confounders, such as intravascular condition of the arteries, time since endovascular intervention, operative complexity, and coagulation abnormalities, may have led to indication bias. Specifically, patients at higher bleeding risk or lower thrombotic risk may have been assigned to the interruption group, whereas those at higher thrombotic risk were more likely to remain in the maintenance group. The difficulty of accurately capturing these patient-level determinants of thrombotic risk reflects an inherent limitation of our database. Our *E*-value analysis suggests that the observed associations are unlikely to be fully explained by unmeasured confounding, particularly when considering the *E*-values for the point estimate. This indicates that relatively strong unmeasured confounding would be required to negate our findings. Nevertheless, considering the E-value for the lower bound of the confidence interval, we acknowledge that moderate unmeasured confounding could still account for the effect, and residual confounding cannot be entirely excluded. Second, our analysis did not account for preoperative thrombotic events in patients who discontinued P2Y12 inhibitors before surgery, and may have thereby underestimated thrombotic risk in the interruption group. Third, the rarity of the targeted outcomes and the limited sample size affected the precision of the RRs, resulting in wide 95% CIs. Fourth, we did not assess low-grade complications defined solely by diagnostic codes, to ensure greater specificity of outcome and clinical relevance by focusing on events requiring therapeutic interventions. This approach consequently limited the evaluation of certain clinically relevant conditions, including stroke or subclinical ischemic events detectable only by imaging studies such as echocardiography. Fifth, we excluded patients receiving anticoagulant therapy during the preoperative period to focus on arterial thrombotic events and reduce confounding. This exclusion may limit the transportability of our findings when applied to more heterogeneous real-world populations, including those at elevated risk for VTE. Sixth, the definition of exposure in this study, discontinuation of antiplatelet therapy for at least 5 days, was based on clinical consensus reported in prior observational studies [24, 25]. Although clinically practical, this definition does not fully reflect the pharmacodynamic diversity among P2Y12 inhibitors. Future studies may explore alternative definitions tailored to each agent, such as the 7-day discontinuation recommended for some irreversible P2Y12 inhibitors. Seventh, although ticlopidine and ticagrelor were excluded due to their limited use and differing clinical profiles in Japan, this exclusion may limit the transportability of our findings to regions where ticagrelor is more commonly used. Finally, we could not identify the direct cause of some deaths in the databases. In some instances, deaths were attributed to CAD exacerbations, but the lack of detailed mortality data hindered robust outcome assessments.

## Conclusions

Discontinuation of antiplatelet therapy more than 5 days before abdominopelvic MIS in patients receiving P2Y12 inhibitors was associated with an increased risk of postoperative CAD, without a clear indication of benefit in terms of bleeding control or mortality. These findings suggest that caution may be warranted regarding discontinuation of antiplatelet therapy before MIS in patients using P2Y12 inhibitors for CAD, as discontinuation might increase the risk of postoperative secondary interventions such as PCI or CABG.

## Supplementary Information

Additional file 1. This file includes supplementary tables, figures, and appendices supporting the findings of the main manuscript. Tables S1–S4. Table S1. Coding definitions for inclusion and exclusion criteria. Table S2. Coding definitions for thrombotic, bleeding, and mortality outcomes. Table S3. Coding definitions for covariates. Table S4. Details of missing data.Figures S1–S6Figures S1–S6. Figures S1–S6. Figure S1. Distributional balance for propensity scores before and after inverse probability of treatment weighting (IPTW) in the main analysis. Figure S2. Standardized mean difference (SMD) plots before and after IPTW in the main and sensitivity analyses. Figure S3. Results of sensitivity analyses. Figure S4. SMD plots before and after weighting in five subgroup cohorts. Figure S5. Subgroup analyses stratified by pelvic and abdominal surgery. Figure S6. E-values and bias plot for risk ratios.

## Data Availability

Due to the nature of this research, participants of this study did not agree for their data to be shared publicly, so supporting data is not available. All data were anonymized prior to analysis. Access to the datasets was governed by data use agreements with JMDC Inc. and HCEI under applicable data protection laws in Japan.

## Abbreviations

ASA: Acetylsalicylic acid
BMI: Body mass index
CABG: Coronary artery bypass grafting
CAD: Coronary artery disease
CI: Confidence interval
DAPT: Dual antiplatelet therapy
IPTW: Inverse probability of treatment weighting
MIS: Minimally invasive surgery
PAD: Peripheral arterial disease
PCI: Percutaneous coronary intervention
RD: Risk difference
RR: Risk ratio
SAPT: Single antiplatelet therapy
SMD: Standardized mean difference
