# Supplementary material for: Association between preoperative interruption of antiplatelet therapy and postoperative thrombotic risk after minimally invasive surgery for abdominopelvic cancer in patients treated with P2Y12 inhibitors
Source: BMC Med. 2026 Jan 16;24:89. doi: 10.1186/s12916-026-04634-0 (PMC12892507; doi:10.1186/s12916-026-04634-0)
Supplement: Supplementary file 1 — Additional file 1. This file includes supplementary tables, figures, and appendices supporting the findings of the main manuscript. Tables S1–S4. Table S1. Coding definitions for inclusion and exclusion criteria. Table S2. Coding definitions for thrombotic, bleeding, and mortality outcomes. Table S3. Coding definitions for covariates. Table S4. Details of missing data.Figures S1–S6Figures S1–S6. Figures S1–S6. Figure S1. Distributional balance for propensity scores before and after inverse probability of treatment weighting (IPTW) in the main analysis. Figure S2. Standardized mean difference (SMD) plots before and after IPTW in the main and sensitivity analyses. Figure S3. Results of sensitivity analyses. Figure S4. SMD plots before and after weighting in five subgroup cohorts. Figure S5. Subgroup analyses stratified by pelvic and abdominal surgery. Figure S6. E-values and bias plot for risk ratios. [file 12916_2026_4634_MOESM1_ESM.docx]

**Additional file 1**

**Title:**

Association between preoperative interruption of antiplatelet therapy and postoperative thrombotic risk after minimally invasive surgery for abdominopelvic cancer in patients treated with P2Y12 inhibitors

**Authors’ Names:**

Masashi Kubota^1,2^, Satomi Yoshida^3^, Takayuki Goto^2^, Toshiki Fukasawa^1^, Takayuki Anno^1^, Gaku Fujiwara^1^, Satoshi Toshiyama^1^, Yoshihide Inayama^1,4^, Takanori Yanai^1^, Takayuki Sumiyoshi^2^, Ryoichi Saito^2^, Takashi Kobayashi^2^, Koji Kawakami^1, *^

**Authors’ Affiliations:**

^1^Department of Pharmacoepidemiology, Graduate School of Medicine and Public Health, Kyoto University, Kyoto, Japan

^2^Department of Urology, Graduate School of Medicine, Kyoto University, Kyoto, Japan

^3^Department of Clinical Medicine, Institute of Medicine, University of Tsukuba, Tsukuba, Japan

^4^Department of Gynecology and Obstetrics, Graduate School of Medicine, Kyoto University, Kyoto, Japan

***Corresponding Author:**

Koji Kawakami, MD, PhD

Department of Pharmacoepidemiology, Graduate School of Medicine and Public Health, Kyoto University, Yoshidakonoecho, Sakyo-ku, Kyoto 606-8501, Japan.

Tel.: +81 75 7539469

Fax: +81 75 7534469

E-mail: kawakami.koji.4e@kyoto-u.ac.jp

ORCID ID: 0000-0002-7477-4071

**Additional file 1 - Index**

| **Supplementary Tables** |  |
| --- | --- |
| **Table S1.** Coding definitions for inclusion and exclusion criteria | Page 3 |
| **Table S2.** Coding definitions for outcomes | Page 8 |
| **Table S3.** Coding definitions for covariates | Page 10 |
| **Table S4.** Details of missing data | Page 13 |
| **Supplementary Figures** |  |
| **Figure S1.** Distributional balance for propensity scores before and after inverse probability treatment weighting in the main analysis | Page 15 |
| **Figure S2.** Standardized mean difference (SMD) plots before and after inverse probability treatment weighting in the main and two sensitivity analyses | Page 16 |
| **Figure S3.** Results of the sensitivity analyses | Page 17 |
| **Figure S4.** Standardized mean difference (SMD) plots before and after weighting in the five subgroup cohorts | Page 18 |
| **Figure S5.** Results of subgroup analyses stratified by pelvic surgery and abdominal surgery. | Page 19 |
| **Figure S6.** E-values and bias plot for risk ratios | Page 20 |
| **Supplementary Appendixes** |  |
| **Appendix 1.** R codes for main analysis | Page 21 |

**Table S1.** Coding definitions for inclusion and exclusion criteria

| **Inclusion criteria** | **Definition** | **Assessment period** |
| --- | --- | --- |
| **P2Y12 Inhibitor** | WHO-ATC code: B01AC04, B01AC22  YJ code: 3399101F1021 | [–28, 0] |
| **Surgery type** | | |
| Colectomy, sigmoidectomy | Procedure code: 150324910, 150442110 | [0, 0] |
| Distal pancreatectomy | Procedure code: 150389310, 150363510, 150389210, 150418310, 150418410 | [0, 0] |
| Distal gastrectomy | Procedure code: 150323510, 150406710 | [0, 0] |
| Hepatic resection | Procedure code: 150348010, 150417810, 150417910, 150441810, 150441910 | [0, 0] |
| Hepatic segmentectomy | Procedure code: 150348110, 150388710, 150388910, 150389010, 150388810, 150441510, 150441610, 150442010, 150442410 | [0, 0] |
| Total hysterectomy | Procedure code: 150379810, 150409810, 150409310 | [0, 0] |
| Total nephroureterectomy | Procedure code: 150326010, 150430510 | [0, 0] |
| Pancreaticoduodenectomy | Procedure code: 150418510, 150389410, 150418710 | [0, 0] |
| Partial nephrectomy | Procedure code: 150325710, 150430410, 150436510 | [0, 0] |
| Proximal gastrectomy | Procedure code: 150377910, 150406910 | [0, 0] |
| Radical prostatectomy | Procedure code: 150326510, 150390310 | [0, 0] |
| Rectal resection | Procedure code: 150325210, 150337810, 150429910, 150430110, 150407210, 150407310 | [0, 0] |
| Rectal amputation | Procedure code: 150337910, 150407410 | [0, 0] |
| Total cystectomy | Procedure code: 150365210, 150403310, 150403410, 150403510, 150407710, 150407510, 150407610 | [0, 0] |
| Total gastrectomy | Procedure code: 150323710, 150429110,150407110 | [0, 0] |
| Total nephrectomy | Procedure code: 150325910, 150389910 | [0, 0] |
| **Cancer type** | | |
| Colon cancer | ICD-10 code: C170, C171, C172, C179, C180, C181, C182, C183, C184, C185, C186, C187, C189 | [–∞, 0] |
| Rectal cancer | ICD-10 code: C19-, C20-, C210, C211 | [–∞, 0] |
| Stomach cancer | ICD-10 code: C160, C161, C162, C163, C164, C165, C166, C169 | [–∞, 0] |
| Prostate cancer | ICD-10 code: C61- | [–∞, 0] |
| Kidney cancer | ICD-10 code: C64- | [–∞, 0] |
| Bladder cancer | ICD-10 code: C670, C671, C672, C673, C674, C675, C676, C677, C679, C680 | [–∞, 0] |
| Upper tract urothelial cancer | ICD-10 code: C65-, C66- | [–∞, 0] |
| Liver cancer | ICD-10 code: C220, C221, C222, C223, C224, C227, C229 | [–∞, 0] |
| Pancreatic cancer | ICD-10 code: C250, C251, C252, C253, C254, C257, C258, C259 | [–∞, 0] |
| Bile duct cancer | ICD-10 code: C240, C241, C248, C249 | [–∞, 0] |
| Endometrial cancer, and cervical cancer | ICD-10 code: C530, C531, C538, C539, C540, C541, C542, C543, C549, C55- | [–∞, 0] |
| **Exclusion criteria** | **Definition** | **Assessment period** |
| Anticoagulant therapy | WHO-ATC code: B01AA03, B01AE07, B01AF01, B01AF02, B01AF03 | [–28, 0] |
| Recurrent cancer | Values indicating 'primary or recurrent cancer' were recorded in the DPC system at hospital admission. | [–28, –1] |
| Metastatic cancer | Values of the 'clinical M1 stage' were recorded in the DPC system at hospital admission. | [–28, –1] |
| Thrombocytopenia | ICD-10 code: D691, D693, D694, D695, D696, D698 | [–∞, –1] |
| Liver cirrhosis | ICD-10 code: K740, K741, K744, K745, K746 | [–∞, –1] |

**Table S2.** Coding definition for outcomes

| **Thrombotic outcomes** | **Definition** | **Assessment period** |
| --- | --- | --- |
| Percutaneous coronary intervention | Procedure code: 150153910, 150374910, 150375010, 150375110, 150145710, 150145810, 150263310, 150284310, 150318310, 150359310, 150375210, 150375310, 150375410, 150443750, 160107550 | [0, 90] |
| Coronary artery bypass graft | Procedure code: 150145910, 150146010, 150302770, 150318410, 150318510 | [0, 90] |
| Ischemic stroke | Procedure code: 150273510, 150301110, 150301210, 150372510, 150380850  WHO-ATC code: B01AD02, B01AD04, B01AE03, N07XX14  YJ code: 3999411G5066 | [0, 90] |
| Peripheral angioplasty | Procedure code: 150153810 | [0, 90] |
| **Bleeding outcomes** | **Definition** | **Assessment period** |
| Endovascular embolization | Procedure code: 150360610 | [0, 90] |
| Open hemostasis surgery | Procedure code: 150160010, 150361410 | [0, 90] |
| Endoscopic hemostasis | Procedure code: 150164850, 150263950, 150199450, 150390010 | [0, 90] |
| Blood transfusion | Procedure code: 150224910, 150286310 | [0, 90] |
| Mortality | Patients with a "death flag" in the diagnosis file, as well as those recorded as "death" in the discharge outcome. | [0, 90] |

**Table S3.** Coding definitions for covariates

| **Covariates** | **Definition** | **Assessment period** |
| --- | --- | --- |
| Age | Age at the date of surgery | [0, 0] |
| Sex | Men, or women | [0, 0] |
| Body mass index (BMI) | Data derived from measured values at hospital admission included in the DPC system.  Category: <18.5 kg/m², 18.5 to <25.0 kg/m², 25.0 to <30.0 kg/m², ≥30 kg/m², or missing | [–27, –1] |
| Smoking, pack-years | Data derived from values obtained through patient interviews at hospital admission, included in the DPC system. | [–∞, –1] |
| Clinical TN stage | Values of the 'clinical T and N stages' were recorded in the DPC system at hospital admission. | [–27, –1] |
| **Comorbidity** | **Definition** | **Assessment period** |
| Coronary artery disease | ICD-10 code: I201, I208, I209, I210, I211, I212, I213, I214, I219, I220, I221, I228, I229, I230, I232, I233, I234, I235, I236, I238, I240, I241, I248, I249, I252 | [–∞, –1] |
| Percutaneous coronary intervention | ・ICD-10 code: Z955  or  ・Procedure code: 150153910, 150374910, 150375010, 150375110, 150145710, 150145810, 150263310, 150284310, 150318310, 150359310, 150375210, 150375310, 150375410, 150443750, 160107550 | [–∞, –1] |
| Coronary artery bypass graft | ・ICD-10 code: Z951  or  ・Procedure code: 150145910, 150146010, 150302770, 150318410, 150318510 | [–∞, –1] |
| Ischemic stroke | ICD-10 code: I630, I631, I632, I633, I634, I635, I636, I638, I639, I64-, I660, I661, I662, I663, I668, I669 | [–∞, –1] |
| Peripheral arterial disease | ICD-10 code: I650, I651, I652, I653, I700, I701, I702, I708, I709 | [–∞, –1] |
| History of diabetic complications | ICD-10 code: E102, E112, E132, E142, E103, E113, E133, E143, E104, E114, E134, E144  WHO-ATC code: A10 | [–∞, –1] |
| Daily P2Y12 inhibitor type and dose (/day) | WHO-ATC code: B01AC04, B01AC22 YJ code: 3399101F1021 | [–28, –28] |
| Number of administered antiplatelets | WHO-ATC code: B01AC04, B01AC22, A01AD05, B01AC06, N02BA01, B01AC56, B01AC23  YJ code: 3399101F1021 | [–28, –28] |
| Daily combined antiplatelets | WHO-ATC code: A01AD05, B01AC06, N02BA01, B01AC56, B01AC23 | [–28, –28] |
| Robot-assisted surgery | Procedure code: 150389910, 150390310, 150406710, 150406910, 150407110, 150407210, 150407310, 150407410, 150407510, 150407610, 150407710, 150409310, 150418310, 150418410, 150430410, 150430510, 150436510, 150441510, 150441610, 150441810, 150441910, 150442010, 150442110, 150442410 | [0, 0] |
| **Maintenance antiplatelets during surgery** | **Definition** | **Assessment period** |
| P2Y12 inhibitor | WHO-ATC code: B01AC04, B01AC22 YJ code: 3399101F1021 | [–4, 0] |
| Acetylsalicylic acid | WHO-ATC code: A01AD05, B01AC06, N02BA01, B01AC56 | [–4, 0] |
| Cilostazol | WHO-ATC code: B01AC23 | [–4, 0] |

**Table S4.** Details of missing data

| **Parameter, N (%)** | **Overall**  **N = 1365** | **Interruption group**  **N = 1157** | **Maintenance group**  **N = 208** |
| --- | --- | --- | --- |
| Age, median | 0 (0) | 0 (0) | 0 (0) |
| Men | 0 (0) | 0 (0) | 0 (0) |
| BMI, median | 104 (7.6) | 97 (8.4) | 7 (3.4) |
| Smoking, pack-years ≥ 20 | 0 (0) | 0 (0) | 0 (0) |
| Hospital size, beds ≥ 500 | 0 (0) | 0 (0) | 0 (0) |
| Clinical T≥3, or N≥1 cancer | 99 (7.3) | 90 (7.8) | 9 (4.3) |
| History of arterial vascular disease | 0 (0) | 0 (0) | 0 (0) |
| CAD (medication only) | 0 (0) | 0 (0) | 0 (0) |
| Post-PCI | 0 (0) | 0 (0) | 0 (0) |
| Post-CABG | 0 (0) | 0 (0) | 0 (0) |
| Ischemic stroke | 0 (0) | 0 (0) | 0 (0) |
| PAD | 0 (0) | 0 (0) | 0 (0) |
| History of diabetic complications | 0 (0) | 0 (0) | 0 (0) |
| P2Y12 inhibitor type and dose | 0 (0) | 0 (0) | 0 (0) |
| Number of combined antiplatelets | 0 (0) | 0 (0) | 0 (0) |
| Type of combined antiplatelets | 0 (0) | 0 (0) | 0 (0) |
| Robot-assisted surgery | 0 (0) | 0 (0) | 0 (0) |
| Surgical site and type | 0 (0) | 0 (0) | 0 (0) |
| Maintenance antiplatelets during surgery | 0 (0) | 0 (0) | 0 (0) |
| **Total number of cases with missing values** | **190 (13.9)** | **174 (15.0)** | **16 (7.7)** |

*Abbreviations: AMI, acute myocardial infarction; BMI, body mass index; CABG, coronary artery bypass graft; CAD, coronary artery disease; PAD, peripheral arterial disease; PCI, percutaneous coronary intervention.*

**Figure S1.** Distributional balance for propensity scores before and after inverse probability treatment weighting in the main analysis


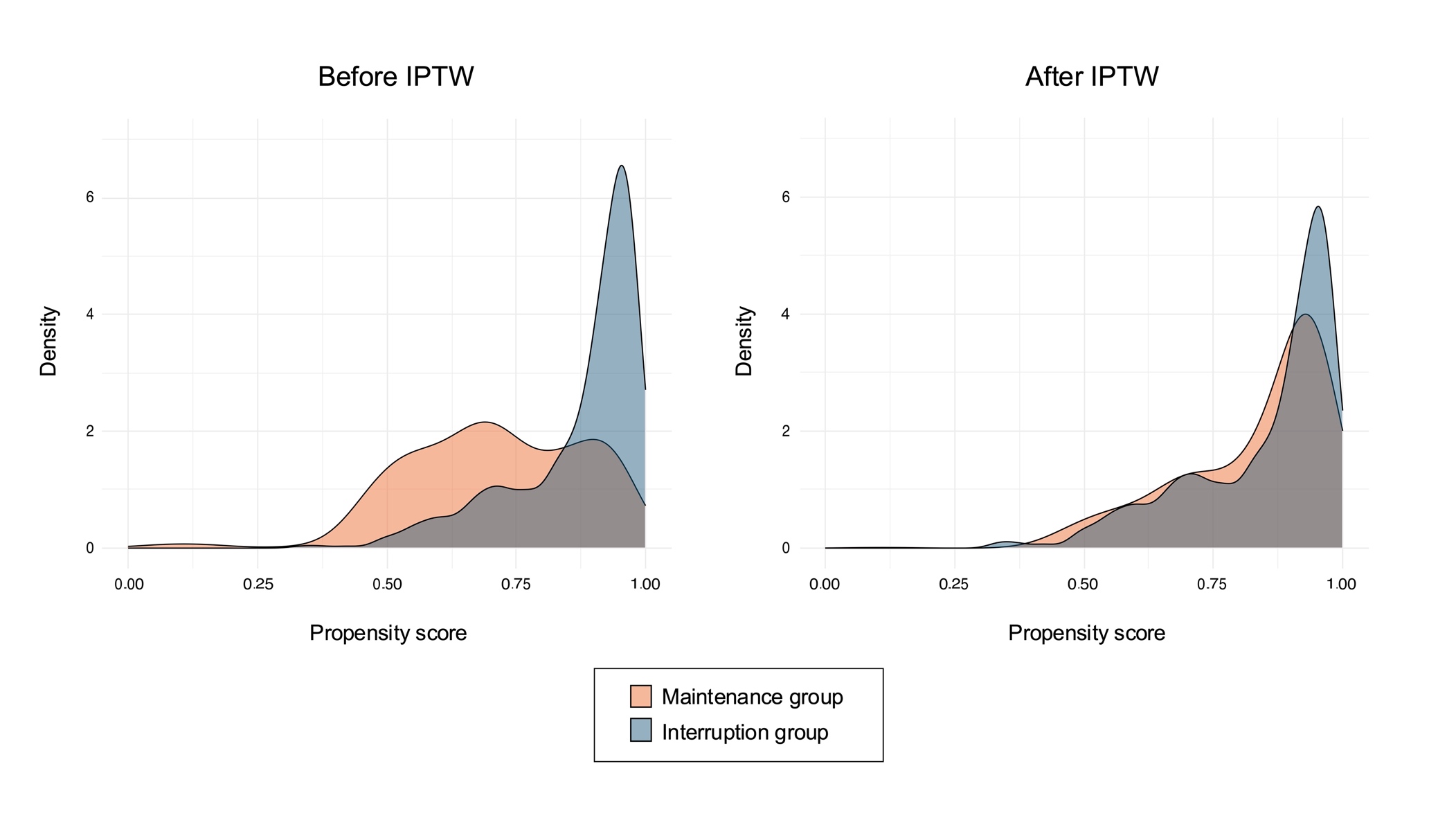


**Figure S2.** Standardized mean difference (SMD) plots before and after inverse

**Figure S3.** Results of the sensitivity analyses

**Figure S4.** Standardized mean difference (SMD) plots before and after weighting in the seven subgroup cohorts

**Figure S5.** Results of subgroup analyses stratified by pelvic surgery and abdominal surgery.

***Pelvic surgery*** includes colectomy, sigmoidectomy, radical prostatectomy, rectal resection, rectal amputation, total cystectomy, and total hysterectomy.

***Abdominal surger****y* includes distal gastrectomy, proximal gastrectomy, total gastrectomy, hepatic resection, hepatic segmentectomy, distal pancreatectomy, total nephroureterectomy, total nephrectomy, and partial nephrectomy.

**Figure S6.** E-values and bias plot for risk ratios

**Appendix 1.** R codes for main analysis

***Propensity Score Estimation and IPTW***

# 1. Propensity Score Estimation Using Logistic Regression

# Estimate the propensity score using a logistic regression model

logistic_model <- glm(group ~ Age + Male_Sex + BMI + Smoking + hospital_size +

T3orN1cancer + CAD_drug_only + PCI + CABG + Ischemic_stroke + PAD +

DM_complication + T2Y12Inhibitor_type_dose + DAPT + TAPT + ASA + cilostazol +

Robot_surgery + Surgery_type,

data = dataset, family = binomial)

# Predict the propensity score (i.e., probability of receiving treatment)

dataset$pscore <- predict(logistic_model, type = "response")

# 2. Calculation of Inverse Probability of Treatment Weights (IPTW)

# Calculate stabilized weights

dataset$Stabilized_Weight <- ifelse(dataset$group == 1,

1 / dataset$pscore,

1 / (1 - dataset$pscore))

# 3. Trimming of Extreme Weights (99th Percentile)

# Calculate the 99th percentile of the weights

percentile_99 <- quantile(dataset$Stabilized_Weight, 0.99)

# Cap weights exceeding the 99th percentile

dataset$Stabilized_Weight <- ifelse(dataset$Stabilized_Weight > percentile_99,

percentile_99,

dataset$Stabilized_Weight)

# Flag trimmed observations

dataset$Trimming <- ifelse(dataset$Stabilized_Weight == percentile_99, 1, 0)

# View the updated dataset

head(dataset)

***Standardized Mean Difference (SMD) Calculation***

# 1. Load the cobalt package (install if not already installed)

if (!require("cobalt")) {

install.packages("cobalt")

}

library(cobalt)

# 2. Calculate standardized mean differences (SMDs)

# a. SMDs before IPTW (unweighted)

smd_result_before <- bal.tab(group ~ Age + Male_Sex + BMI + Smoking + hospital_size +

T3orN1cancer + CAD_drug_only + PCI + CABG + Ischemic_stroke + PAD +

DM_complication + T2Y12Inhibitor_type_dose + DAPT + TAPT + ASA +

cilostazol + Robot_surgery + Surgery_type,

data = dataset)

# b. SMDs after IPTW (weighted)

smd_result_after <- bal.tab(group ~ Age + Male_Sex + BMI + Smoking + hospital_size +

T3orN1cancer + CAD_drug_only + PCI + CABG + Ischemic_stroke + PAD +

DM_complication + T2Y12Inhibitor_type_dose + DAPT + TAPT + ASA +

cilostazol + Robot_surgery + Surgery_type,

data = dataset, weights = dataset$Stabilized_Weight, method = "weighting")

# 3. Create a data frame containing SMDs before and after weighting

smd_data <- data.frame(

Covariate = rownames(smd_result_before$Balance),

SMD_before = smd_result_before$Balance$Diff.Un,

SMD_after = smd_result_after$Balance$Diff.Adj

)

# View the result

head(smd_data)

***Estimating Risk Ratios and Risk Differences Using Weighted Regression***

# 1. Load required packages (install if not already available)

if (!require("sandwich")) install.packages("sandwich", dependencies = TRUE)

if (!require("lmtest")) install.packages("lmtest", dependencies = TRUE)

library(sandwich)

library(lmtest)

# 2. Define a function to perform weighted Poisson regression and risk difference estimation

run_weighted_models <- function(data, outcome) {

# Default result in case of errors

default_result <- data.frame(

Outcome = outcome,

RR = NA,

`95% CI Lower (RR)` = NA,

`95% CI Upper (RR)` = NA,

`p-value (RR)` = NA,

RD = NA,

`95% CI Lower (RD)` = NA,

`95% CI Upper (RD)` = NA,

`p-value (RD)` = NA,

Error = NA

)

# Ensure the outcome variable is binary (0/1)

if (!all(data[[outcome]] %in% c(0, 1), na.rm = TRUE)) {

default_result$Error <- "Outcome must be binary (0/1)"

return(default_result)

}

# Remove rows with missing values in the outcome variable

data <- data[!is.na(data[[outcome]]), ]

# Ensure the treatment group variable is binary

if (!all(data$group %in% c(0, 1), na.rm = TRUE)) {

default_result$Error <- "Group must be binary (0/1)"

return(default_result)

}

# 2a. Poisson regression for Risk Ratio (RR)

model_rr <- tryCatch(

glm(as.formula(paste(outcome, "~ group")),

data = data,

family = poisson(link = "log"),

weights = data$Stabilized_Weight),

error = function(e) return(NULL)

)

if (is.null(model_rr)) {

default_result$Error <- "Poisson model did not converge"

return(default_result)

}

robust_se_rr <- coeftest(model_rr, vcov = vcovHC(model_rr, type = "HC0"))

RR <- exp(robust_se_rr["group", "Estimate"])

lower_CI_RR <- exp(robust_se_rr["group", "Estimate"] - 1.96 * robust_se_rr["group", "Std. Error"])

upper_CI_RR <- exp(robust_se_rr["group", "Estimate"] + 1.96 * robust_se_rr["group", "Std. Error"])

p_value_RR <- robust_se_rr["group", "Pr(>|z|)"]

# --- Risk Difference (RD) ---

# Try identity-link binomial; if it errors, fails to converge, or yields out-of-bounds fits,

# fall back to a weighted Linear Probability Model (LPM) with robust SEs.

warn_msg <- NULL

model_rd <- tryCatch(

withCallingHandlers(

glm(as.formula(paste(outcome, "~ group")),

data = data,

family = binomial(link = "identity"),

weights = data$Stabilized_Weight),

warning = function(w) { warn_msg <<- conditionMessage(w); invokeRestart("muffleWarning") }

),

error = function(e) NULL

)

# Non-convergence / bad-fit criteria

bad_fit <- is.null(model_rd) ||

isFALSE(isTRUE(model_rd$converged)) ||

any(!is.finite(fitted(model_rd))) ||

any(fitted(model_rd) < 0 | fitted(model_rd) > 1) ||

(is.character(warn_msg) && grepl("did not converge|fitted probabilities", warn_msg, ignore.case = TRUE))

if (bad_fit) {

# ---- Fallback: weighted LPM (lm) + robust SEs ----

model_lpm <- lm(as.formula(paste(outcome, "~ group")),

data = data, weights = data$Stabilized_Weight)

robust_se_rd <- coeftest(model_lpm, vcov = vcovHC(model_lpm, type = "HC0"))

} else {

# ---- Identity-link binomial succeeded ----

robust_se_rd <- coeftest(model_rd, vcov = vcovHC(model_rd, type = "HC0"))

}

# Extract the coefficient for 'group' robustly (handles "group" or "group1", etc.)

coef_row <- grep("^group", rownames(robust_se_rd), value = TRUE)

RD <- robust_se_rd[coef_row, "Estimate"]

SE <- robust_se_rd[coef_row, "Std. Error"]

lower_CI_RD <- RD - 1.96 * SE

upper_CI_RD <- RD + 1.96 * SE

p_value_RD <- robust_se_rd[coef_row, 4]

# Combine results into a data frame

result <- data.frame(

Outcome = outcome,

RR = RR,

`95% CI Lower (RR)` = lower_CI_RR,

`95% CI Upper (RR)` = upper_CI_RR,

`p-value (RR)` = p_value_RR,

RD = RD,

`95% CI Lower (RD)` = lower_CI_RD,

`95% CI Upper (RD)` = upper_CI_RD,

`p-value (RD)` = p_value_RD,

Error = NA

)

return(result)

}

# 3. Define list of binary outcomes to analyze

outcomes <- c(

"Thrombotic_complications",

"PCI_or_CABG",

"Strokes",

"Peripheral_Angioplasty",

"Hemostasis_procedure",

"Endovascular_embolization",

"Open_hemostasis",

"Endoscopic_hemostasis",

"Blood_transfusion",

"Mortality",

"Combined_vascular_events"

)

# 4. Apply the analysis function to each outcome

results_list <- lapply(outcomes, function(o) {

cat("\nProcessing outcome:", o, "\n")

run_weighted_models(dataset, o)

})

# 5. Combine results into a single data frame

final_results <- do.call(rbind, results_list)

# 6. Display the final results

print(final_results)

***Calculation of E-values and Bias Plot Visualization for Relative Risks***

# Load libraries

library(dplyr)

library(EValue)

# Calculate E-values and add as new columns

bosu <- bosu %>%

rowwise() %>%

mutate(

# evalues.RR takes est, lo, hi, true

evalue_calc = list(as.list(evalues.RR(est = RR, lo = lo, hi = hi, true = 1))),

Evalue_point = evalue_calc[["point"]], # Point estimate-based E-value

Evalue_lower = evalue_calc[["lower"]] # CI lower-bound-based E-value

) %>%

ungroup() %>%

select(-evalue_calc)

head(bosu)

# Function to create a bias plot for a given RR

plot_bias <- function(rr_value, xmax = 20) {

bias_plot(rr_value, xmax = xmax)

}

# Save bias plot as SVG

svg(filename = "Outcome_biasplot.svg", width = 6, height = 6) # width & height in inches

bias_plot(rr_value, xmax = 20) # your bias plot

dev.off()

***IPTW-weighted cumulative incidence function (CIF) with death as competing risk***

# ===============================

# Weighted CIF (Competing Risks: Death as competing event)

# ===============================

# Requirements:

# - status coding: 0 = censored, 1 = event of interest, 2 = death (competing event)

# - 'group' is a binary indicator (0/1) to be contrasted

# - 'weight' is a (stabilized) IPTW

#

# Packages:

# install.packages(c("dplyr","prodlim"))

library(dplyr)

library(prodlim)

# ---- Function ----

plot_weighted_cif <- function(data,

time_col = "time",

status_col = "status_col", # 0=censor, 1=event, 2=death

group_col = "group",

weight_col = "Stabilized_Weight",

group_labels = c("Group 0","Group 1"),

cause_of_interest = 1,

xlab = "Time (days)",

ylab = "Cumulative incidence",

line_width = 2,

colors = c("gray25","firebrick"), # order: group 0, group 1

xlim = NULL, ylim = NULL,

x_ticks = NULL, # e.g., seq(0, 90, by = 10)

add_title = NULL,

svg_file = NULL, svg_width = 6, svg_height = 5) {

# Prepare data (no trimming assumed; user handles it upstream if desired)

df <- data %>%

mutate(

.grp = factor(.data[[group_col]], levels = c(0,1), labels = group_labels),

.w = .data[[weight_col]],

.t = .data[[time_col]],

.s = .data[[status_col]]

) %>%

filter(is.finite(.w), .w > 0)

# Report raw and weighted sample sizes

cat("Sample sizes by group (raw vs weighted sum):\n")

print(

df %>%

group_by(.grp) %>%

summarise(n_raw = dplyr::n(),

n_wt = sum(.w),

mean_wt = mean(.w),

sd_wt = sd(.w),

.groups = "drop")

)

# Fit weighted CIF

fit_cr <- prodlim::prodlim(

formula = prodlim::Hist(.t, .s) ~ .grp,

data = df,

caseweights = df$.w

)

# Open SVG device if requested

if (!is.null(svg_file)) {

svg(filename = svg_file, width = svg_width, height = svg_height)

on.exit(dev.off(), add = TRUE)

}

# Plot CIF for event of interest

plot(fit_cr,

type = "cuminc",

cause = cause_of_interest,

xlab = xlab,

ylab = ylab,

lwd = line_width,

col = colors,

legend = TRUE,

xlim = xlim,

ylim = ylim)

if (!is.null(x_ticks)) axis(1, at = x_ticks)

if (!is.null(add_title)) title(main = add_title)

invisible(fit_cr)

}

# ---- Example usage ----

# plot_weighted_cif(

# data = bosu,

# time_col = "time",

# status_col = "status_col", # 0=censor, 1=event, 2=death

# group_col = "group",

# weight_col = "Stabilized_Weight",

# group_labels = c("Group 0","Group 1"),

# cause_of_interest = 1,

# colors = c("gray25","firebrick"),

# xlim = c(0, 90), ylim = c(0, 0.05),

# x_ticks = seq(0, 90, by = 10),

# add_title = "Cumulative Incidence Function (Event of Interest; Death as competing event)",

# svg_file = "CIF_plot.svg", svg_width = 6, svg_height = 7

# )
